# Inferior and precordial ST-segment elevation myocardial infarction due to large wrap-around left anterior descending artery occlusion

**DOI:** 10.1093/omcr/omad038

**Published:** 2023-04-20

**Authors:** Hiroto Tamura, Yohei Tobetto, Akiho Seno, Koichi Kishi

**Affiliations:** Department of Cardiology, Tokushima Red Cross Hospital, Tokushima, Japan; Department of Cardiology, Tokushima Red Cross Hospital, Tokushima, Japan; Department of Cardiology, Tokushima Red Cross Hospital, Tokushima, Japan; Department of Cardiology, Tokushima Red Cross Hospital, Tokushima, Japan

## Abstract

Left anterior descending artery (LAD) occlusion normally develops into precordial ST-segment elevation; however, we describe a case of a 50-year-old man with inferior and precordial ST-segment elevation myocardial infarction that resulted from proximal occlusion of the wrap-around LAD perfusing the anterior and inferior wall. We performed early and prompt reperfusion and were able to save the patient without any complications. A wrap-around LAD perfuses a large myocardial area; therefore, this type of coronary occlusion causes severe myocardial damage and has a poor prognosis. The rates of new-onset heart failure and heart failure rehospitalization are also high. Simultaneous inferior and precordial ST-segment elevations on electrocardiography suggest acute myocardial infarction with a wrap-around LAD lesion, which requires prompt revascularization. More careful medications for heart failure and follow-up should be also required even after discharge in such case.

## INTRODUCTION

Generally, left anterior descending artery (LAD) occlusion results in acute myocardial infarction (AMI) with ST-segment elevation in the precordial leads and ST-segment depression in the inferior leads. Inferior leads are reciprocal to precordial leads; therefore, simultaneous inferior and precordial ST-segment elevation in myocardial infarction is rare. Here, we describe inferior and precordial ST-segment elevation myocardial infarction due to a large wrap-around LAD occlusion.

## CASE REPORT

**Figure 1:**
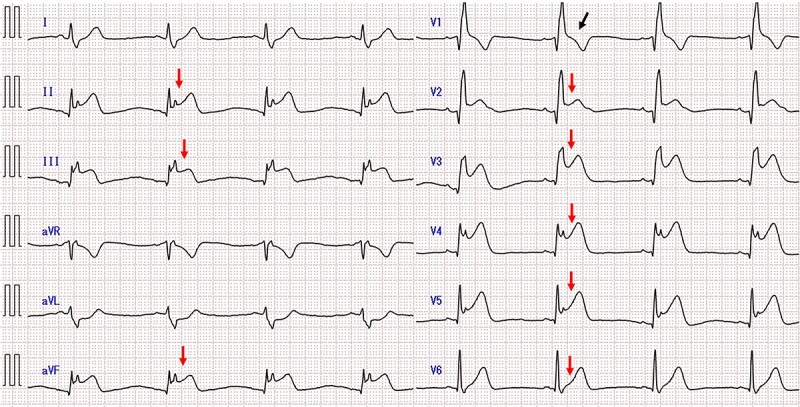
Admission ECG showing ST-segment elevation in leads II, III, aVf and V2-6 (red arrows) along with CRBBB (black arrow).

**Figure 2:**
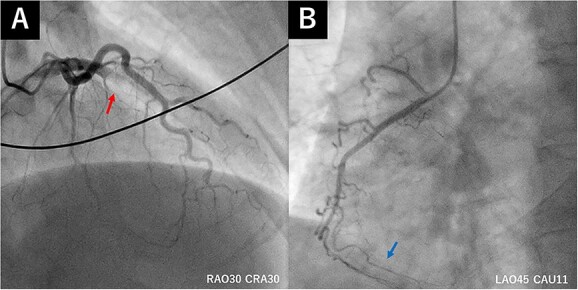
(**A**) Initial CAG showing proximal LAD occlusion (red arrow). (**B**) Initial CAG showing a small RCA (blue arrow).

**Figure 3:**
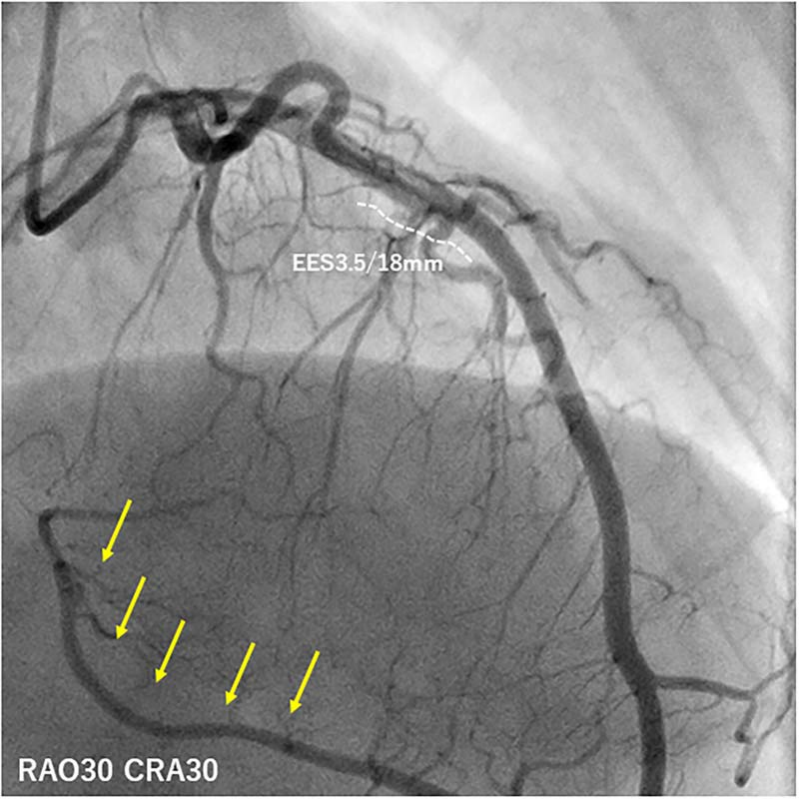
CAG immediately after PCI showing a wrap-around LAD that perfuses the inferior myocardial wall (yellow arrows).

**Figure 4:**
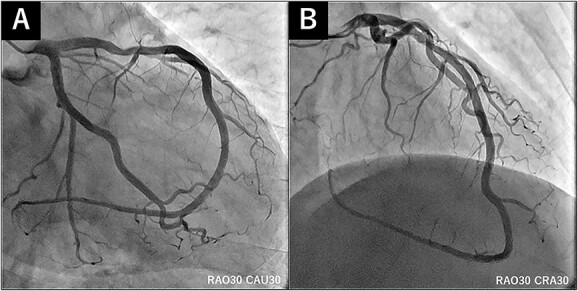
(**A**) and (**B**) CAG 3 years after discharge showing no restenosis.

**Figure 5:**
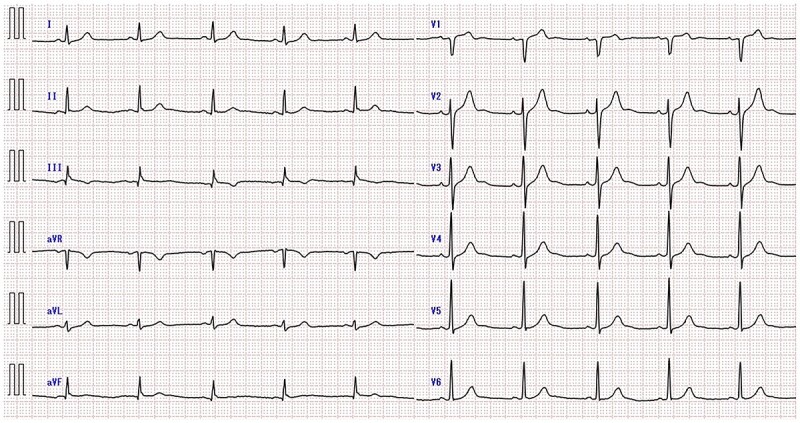
ECG 3 years after discharge showing disappearance of CRBBB and improvement of ST-segment elevation.

A 50-year-old man had sudden chest pain. One hour later, he was admitted to our emergency department. The patient’s past medical history included hyperuricemia, ureterolithiasis and current smoking. On admission, his blood pressure was 98/58 mmHg; pulse rate, 51 beats/min and oxygen saturation, 96% (mask, 3 L). The patient had no murmurs or respiratory rales and was experiencing cold sweats. Electrocardiography (ECG) showed ST-segment elevation in leads II, III, aVf and V2-6 (Fig. 1) along with complete right-bundle branch block (CRBBB). Echocardiography revealed moderate left ventricular dysfunction with anteroseptal and inferior wall motion abnormalities. Initially, we expected simultaneous occlusion of the right coronary artery (RCA) and LAD. Emergency coronary angiography (CAG) showed proximal LAD occlusion (Fig. 2A), a normal left circumflex artery and a small RCA (Fig. 2B). Emergency percutaneous coronary intervention (PCI) of the LAD was performed. The thrombus was aspirated using an aspiration catheter (Thrombuster, KANEKA, Japan) after wire crossing. An everolimus-eluting stent (EES: 3.5 × 18 mm) was placed. During the procedure, the patient developed ventricular fibrillation. We performed defibrillation four times, and his ECG returned to sinus rhythm. CAG after successful stenting revealed large LAD wrapping around the apex to the inferior wall (Fig. 3). Immediately after PCI, we inserted an intra-aortic balloon pump (IABP) for continuation of cardiac shock. The door-to-balloon time was approximately 40 min. While creatinine kinase (CK) levels peaked at 12 079 U/L (normal: 40–200 U/L), CK-MB peaked at 220 U/L (normal: <25 U/L). The patient’s postoperative course was favorable. On day 3 post admission, the IABP was removed for hemodynamic stabilization. Heart failure did not develop during hospitalization. On day 11 postadmission, the patient was discharged without complications. He was able to return to work. After 3 years, CAG revealed no restenosis (Fig. 4A and B), and ECG showed disappearance of the CRBBB and improvement in ST-segment elevation (Fig. 5). Echocardiography revealed residual anteroseptal wall motion abnormalities; however, the ejection fraction was restored to 64%. During the several years following treatment, the patient experienced no further cardiovascular events.

## DISCUSSION

This was a case of a patient who developed AMI with inferior and precordial ST-segment elevation. CAG revealed an occlusion of only the LAD, which ran around the apex to the inferior wall and perfused both the inferior and anterior myocardium. An LAD that perfuses at least one-fourth of the inferior left ventricular wall in the right cranial oblique, is known as a wrap-around LAD [1]. Bozbeyoğlu *et al*. reported 88 patients (34.3%) with wrap-around LAD and 29 patients (11.5%) with ST-elevation in inferior leads among 259 patients with anterior AMI. Among the 29 patients, 13 patients (44.8%) had distal occlusion of wrap-around LAD, which was related to the cause of anterior AMI with ST-elevation in inferior leads [1]. It results in a minor anterior myocardial injury, small reciprocal inferior ST-change and inferior myocardial ischemia [2]. Although CAG revealed proximal occlusion of LAD in this case, the ECG of our patient revealed both inferior and precordial ST-segment elevation. This was a relatively rare case, and we considered the following: the patient had a small RCA and thus, LAD perfused a significant portion of the inferior myocardium. There was a larger change of the ST-elevation than that of ST-depression inferior segments. This type of LAD perfuses a larger portion of the myocardium; therefore, wrap-around LAD occlusion may result in severe myocardial damage compared with that of non-wrap-around LAD occlusion [3]. Kobayashi *et al.* reported a high mortality and morbidity in patients with occluded wrap-around LAD [4]. The rates of new-onset heart failure and heart failure rehospitalization are also high. Medications should be managed and careful follow-up is required, even after discharge. Our patient’s ECG also showed CRBBB, which resulted from an anterior myocardial infarction involving the interventricular septum. Wong *et al*. reported that the right-bundle branch traverses the intraventricular septum toward the cardiac apex [5]. In this case, the occluded lesion was proximal to a large septal branch. We considered that the proximal wrap-around LAD occlusion resulted in ischemia of the septum and the right-bundle branch and development of CRBBB. A poor prognosis of ST-segment elevation myocardial infarction with CRBBB has been reported [6]. The patient was saved due to early and prompt reperfusion. The possibility of wrap-around LAD lesions in simultaneous inferior and precordial ST-segment elevations should be considered. Large infarct area and high incidence of heart failure indicate a poor prognosis. Clinicians should note the need for rapid reperfusion and more careful medication for heart failure in such cases.

## Data Availability

All relevant data are within the manuscript.
